# Amplification of typhoon-generated near-inertial internal waves observed near the Tsushima oceanic front in the Sea of Japan

**DOI:** 10.1038/s41598-023-33813-9

**Published:** 2023-06-02

**Authors:** Yusuke Kawaguchi, Itsuka Yabe, Tomoharu Senjyu, Akie Sakai

**Affiliations:** 1 Atmosphere and Oceanic Research Institute, The University of Tokyo, Chiba, 277-8564 Japan; 2 Research Institute for Applied Mechanics, Kyushu University, Fukuoka, 816-8580 Japan; 3 Interdisciplinary Graduate School of Engineering Sciences, Kyushu University, Fukuoka, 816-8580 Japan

**Keywords:** Physical oceanography, Natural hazards, Ocean sciences

## Abstract

It is not fully understood how near-inertial kinetic energy (NIKE) is spatially distributed near Tsushima oceanic front (TOF) as a typhoon travels across the region. Underneath TOF, a year-round mooring covering a major part of water column was implemented in 2019. During summer, three massive typhoons (*Krosa*, *Tapah*, and *Mitag*) consecutively traversed the frontal area and delivered a substantial amount of NIKE into surface mixed layer. According to a mixed-layer slab model, NIKE was widely distributed near the cyclone’s track. The mooring observation exhibited the vertical distribution and pathways of surface-generated NIKE in response to the successive typhoon events. According to the modal decomposition, first three modes mostly explain the NIKE’s elevations following the typhoon events. According to ray-tracing experiments based on the internal-wave theory, large-scale near-inertial waves (NIWs) rapidly descend to a depth greater than 1000 m, while mesoscale NIWs slowly descend and rarely reached beyond the main pycnocline. Following the passage of *Tapah*, a profound energy mass was found nearly stationary at shallow depths coincident with vertical shear of geostrophic current. We infer that the descending rate of NIWs fell and then they were amplified through the energy conservation when the waves came from the north side of TOF.

## Introduction

In summer and fall, massively developed tropical cyclones, aka typhoons, occasionally pass over the sea and result in an excess of momentum and kinetic energy into the surface water^1–3^. In south-east and east Asian countries, the typhoon events physically damage buildings and infrastructures by gusty winds and/or storm surges if they land on the ground or approach the shore regions^4^. However, it is not well known how kinetic energy of typhoons can be spatially transferred and distributed through the ocean interior as it goes across the sea. Numerous wave kinetic energy-related questions remain to be resolved. For example, it has been challenging to fully pursue the typhoon-induced internal waves travelling down to the deepest level of the sea (e.g., Kawaguchi and Yabe, unpublished; included in supporting materials). Accordingly, many previous studies deduced the paths of internal waves indirectly from disconnected pieces of near-inertial oscillations in current records^5,6^.

The typhoon’s wind forcing at the ocean surface causes an upwelling in the upper layer near its center due to the diverging Ekman transport (the so-called Ekman suction), veering to the right angle of surface wind stress in the northern hemisphere^7^. In consequence, the vertical displacement of water near the base of surface mixed layer (SML) is excited, and its information radiates to the environment waters as freely propagating internal waves, especially with an emphasis of the inertial period^2,8^.

**Figure 1:**
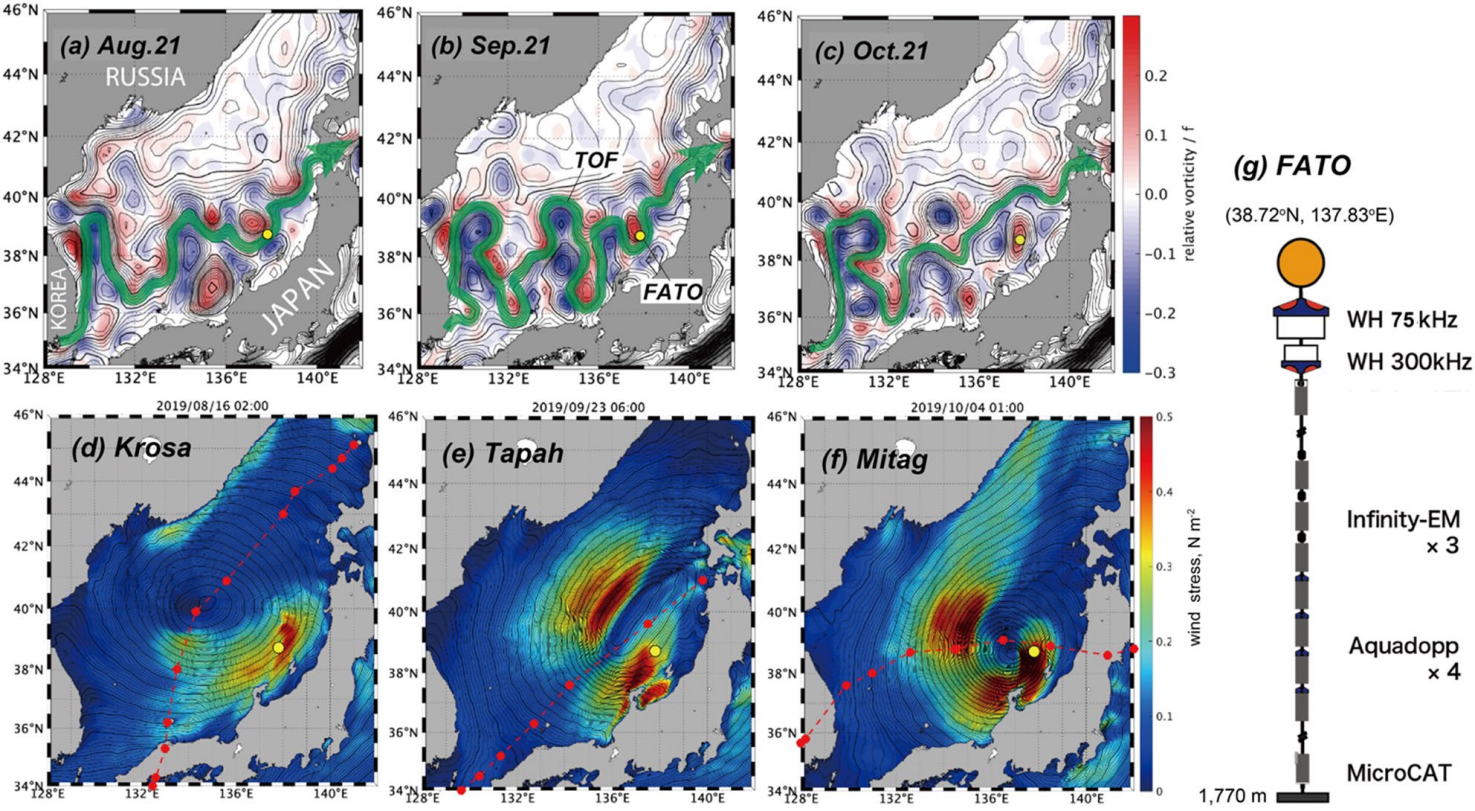
Satellite-based relative vorticity anomaly (RVA) outlined by sea surface height (SSH) (black contour, interval = 2 cm), with a yellow circle for the FATO station: (**a**) August 21, (**b**) September 21, and (**c**) October 21 in 2019. Bold green curve shows the streamline along the Tsushima Oceanic Front (TOF). (**d**–**f**) Snapshots of sea level pressure (isolines) and magnitude of surface wind stress (colors) during passages of *Krosa*, *Tapah*, and *Mitag*, respectively. Red curves with red dots show approximate paths of the typhoon migration, and its location at every 6 h. (**g**) Schematics of the mooring system deployed at the FATO station.

The Tsushima oceanic front (TOF) is prone to meandering and creating mesoscale features with a significant relative vorticity anomaly (RVA) in the upper layer^9,10^ (Fig. 1a–c). It is known that the negative RVA body can lower the minimum frequency of internal wave propagation from the Coriolis frequency, ${\omega }_{\text{min}}=f(\theta )$, to the effective Coriolis frequency, ${f}_{e}=f(\theta ) + {\zeta }_{g}/2$, where $\theta$ is latitude, ${\zeta }_{g}=\frac{\partial {V}_{g}}{\partial x}-\frac{\partial {U}_{g}}{\partial y}$ is the relative vorticity with ${U}_{g}$ and ${V}_{g}$, respectively, as east (x) and northward (y) components of the geostrophic current ${{\varvec{U}}}_{{\varvec{g}}}$^11–14^. From their mooring observation near the TOF, Kawaguchi et al.^15^ demonstrated that a warm-core eddy with negative RVA captured near-inertial kinetic energy (NIKE) associated with a fast-travelling “bomb” cyclone over the sea during summer of 2015. Its NIKE was transferred to multiple inertial frequencies due to non-linear interactions amid the trapped waves. Intriguingly, the same cyclone in summer 2015 was also observed to cause profound turbulent mixing within another mesoscale eddy located in the opposite (i.e., Russian) side of the same ocean^16^.

The propagation of NIKE can be restricted not only by the RVA body but also by vertical shear of the geostrophically balanced baroclinic current. That is, the lower propagation limit can be more strictly expressed as follows:

1 $${\omega }_{\text{min}}=f\sqrt{1+\frac{{\zeta }_{g}}{f}-{Ri}_{g}^{-1}}$$

where ${Ri}_{g}=\frac{{N}^{2}}{{\left(\frac{\partial {U}_{g}}{\partial z}\right)}^{2}+{\left(\frac{\partial {V}_{g}}{\partial z}\right)}^{2}}$ is the gradient Richardson number; ${N}^{2}=-\frac{g}{\rho }\frac{\partial \rho }{\partial z}$ is the buoyancy frequency; $g$ the gravitational acceleration; $z$ is vertical axis pointing upward; and $\rho$ is the density of seawater, which represents the inclination of isopycnal interface^17^. According to their shipboard observations of internal waves and microscale turbulence in the TOF region, Kawaguchi et al. (2021)^18^ demonstrated that the sloping isopycnals created geostrophic shear and limited the propagation of near-inertial waves toward the deeper layers. From a viewpoint of the wave kinetic energy distribution under the sea, it is worth evaluating the roles and impacts of the baroclinicity of the TOF as a function of the typhoon-induced near-inertial waves.

In the regions proximate to the Korean Peninsula, the previous studies revealed that the tidal forcing dominantly generated the internal waves and influenced the local turbulent mixing^19,20^. In the central part of Sea of Japan, the barotropic tidal currents are negligibly small^6,15,18,21^ and are less likely subject to critical corruptions in the current data records by tides. In this sense, the TOF region is an ideal domain for the study of the interaction between oceanic front and wind-induced near-inertial waves.

Within the framework of the FRA-AORI Tsushima Warm Current Observatory (FATO) project, we implemented a year-round mooring observation of water current near the TOF in 2019‒2020. To cover the entire depth where the surface generated near-inertial waves possibly travelled, we installed two sets of acoustic Doppler current profilers (ADCPs) and seven sets of single-point current meters (Fig. 1g) (“Data and methods”). In this paper, we analyzed the moored horizontal current with special focuses on the vertical distribution of NIKE, associated with the recurrent passages of massive typhoons. For the identification of the neighboring sources of near-inertial waves, we computed NIKE generated by the respective typhoon events near surface level across the entire Sea of Japan via the mixed layer slab model^3^. Based on the classical theory of the internal-wave dispersion^11^, the ray-tracing experiments were also conducted to interpret the distribution and vertical transfer of the observed signatures of NIKE. The satellite-based geostrophic currents were depicted as the supporting information of the TOF axis and neighboring mesoscale structures.

## Results

### Typhoon events in summer 2019

Between August and October, three massive typhoons, named *Krosa*, *Tapah*, and *Mitag*, successively or consecutively passed over the Sea of Japan and above the TOF (Table 1; Fig. 1d–f). The observed maximum wind speeds were 40, 35, and 40 m s^−1^, while the minimum pressures near the center were 965, 970, and 965 hPa, respectively. All the typhoons were associated with sufficiently warm surface temperatures in the tropical latitudes (10°–20°N) of the North Pacific Ocean. As for their trajectories, *Krosa* travelled through the middle of the Sea of Japan at a mean speed of 8.3 m s^−1^. *Tapah* went nearly straightforward along the main island of Japan at 11.8 m s^−1^. *Mitag* drew a crooked trajectory in clockwise direction through the southern part of the sea, at a mean travelling speed of 8.8 m s^−1^.

**Table 1:** Summary of typhoons passing over the Sea of Japan in summer 2019.

Name of typhoon	*Krosa* (#10)	*Tapah* (#17)	*Mitag* (#18)
Min pressure, hPa	965	970	965
Max wind speed, m s^−1^	40	35	40
Mean travelling speed (${V}_{\text{cyc}}$), m s^−1^	8.3	11.8	8.8
Periods in 2019	August 15–17	September 22–24	October 3–5

Numbers in parenthesis show the identifications given by JMA.

### Inertial wave kinetic energy input in surface mixed layer

**Figure 2:**
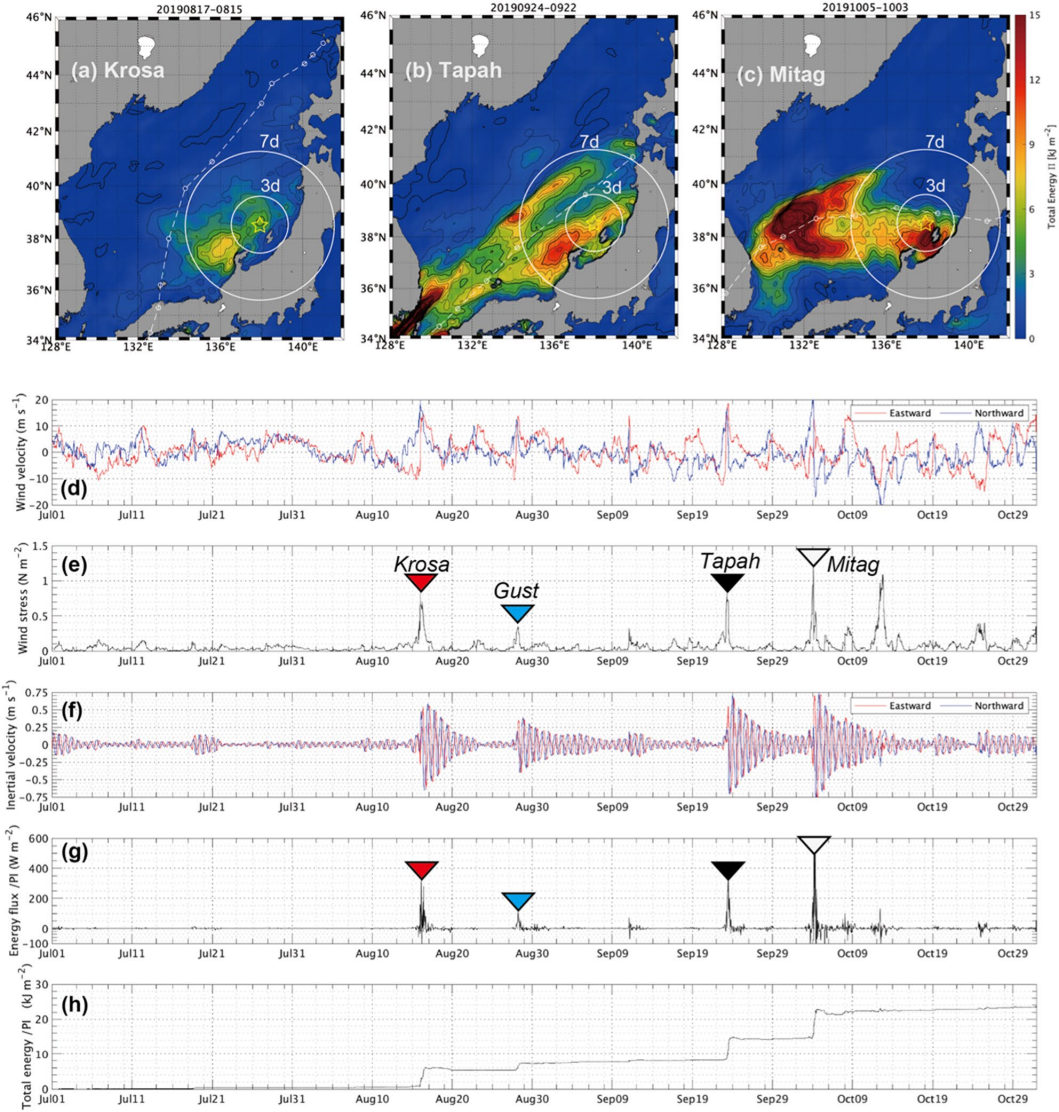
Horizontal map of a 2-d cumulative near-inertial kinetic energy from the slab model during respective passages of (**a**) *Krosa*, (**b**) *Tapah*, and (**c**) *Mitag*. White circles show accessible ranges for near-inertial waves for periods of three and seven days, which are estimated based on its horizontal group velocity (see Fig. 3e). A yellow star shows location of the FATO station. White dashed curves show a track of typhoon’s migration, with a small circle the position at every six hours. Time-series of the slab model simulations at the FATO station: (**d**) surface wind velocity, (**e**) magnitude of wind stress, (**f**) predicted inertial current, (**g**) surface wave kinetic energy flux ($\Pi$), and (**h**) cumulative wave kinetic energy ($\int \mathrm{\Pi dt}$). In (**d**–**f**), eastward and northward components are shown by red and blue, respectively. In (**e**) and (**g**), typhoon events of *Krosa*, *Tapah*, and *Mitag* are indicated by inverted triangles, respectively, in red, black, and white, and the gusty event in blue.

The typhoons-delivered NIKE into SML was estimated from the slab model (see “Data and methods”). In Fig. 2, the cumulative NIKE input (Π) along with the cyclone’s track is illustrated on the geographical map for each event.

The passage of *Krosa* produced a relatively small NIKE input, where the only noticeable patch was found on the right-hand side of the cyclone’s track, centered at the geographical point (38°N, 136°E), whose magnitude was roughly Π = 7 kJ m^−2^ (Fig. 2a). *Tapah* delivered the maximum amount of NIKE at Π = 10–12 kJ m^−2^, relatively evenly distributed at both sides of the cyclone’s track (Fig. 2b). The maximum energy input was spotted roughly 100 km apart from the track, plausibly coincident to the core radius of the cyclone, where the tangential wind speed became greatest. *Mitag* produced an uneven distribution of the NIKE input (Fig. 2c). In the western half domain of the Sea of Japan, approximately along the cyclone track or slightly in its left-hand side, the greatest energy input can be found at the geographical point (39°N, 131–132°E), excessing Π = 15 kJ m^−2^ in the neighborhood. In the eastern domain, *Mitag* provided a relatively moderate NIKE input, in particular, at the right-hand side of its migration track^1^. The maximum surface energy input was found in the small region between the Noto Peninsula and the Sado Island, whose magnitude was approximately Π = 13–15 kJ m^−2^.

At the nearest grid from the FATO station, the slab model simulations provided the accurate predictions of the inertial oscillation generation in the SML following the recurrent atmospheric events (Fig. 2d–h). The model predicted that there were outstanding oscillatory events in the SML in response to the three typhoon passages (Fig. 2e,f). During the passages of *Krosa*, *Tapah*, and *Mitag*, the SML was estimated to receive the net NIKE amounts of (i.e., $\int\Pi dt$) of 5.5, 6.5, and 9 kJ m^−2^, respectively (Fig. 2h). Following the three typhoon events, the slab model indicated that the SML inertial oscillation built up to be 0.5, 0.7, and 0.8 m s^−1^, respectively, in the maximum amplitude of horizontal velocity (Fig. 2f). In addition to the typhoon events, the slab model showed that the transient gusty event that occurred in between *Krosa* and *Tapah* added a certain amount of NIKE (~ 2.0 kJ m^−2^) into the SML, generating an oscillation amplitude of 0.3 m s^−1^ (blue inverted triangle in Fig. 2).

### Hydrographic and mesoscale features

**Figure 3:**
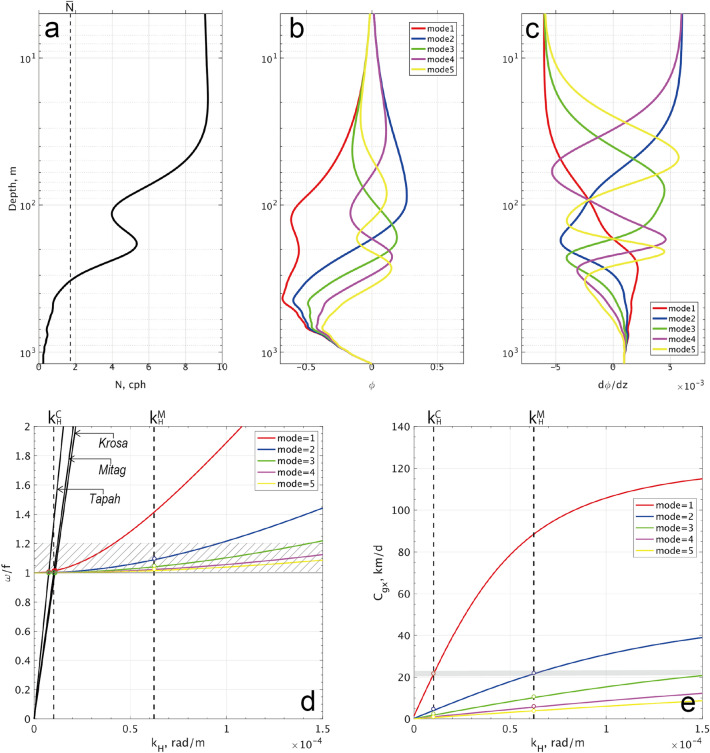
(**a**) Mean vertical profile of $N(z)$ taken on July 20; (**b**, **c**) vertical mode solutions of vertical and horizontal velocities, respectively, $\phi_n$ and $\frac{\partial {\phi_n}}{\partial {z}}$ for $n =$ 1, 2, 3, 4, and 5; the dispersion relation of internal waves for $n =$ 1–5: (**d**) $\omega$ vs. $k_H$, and (**e**) $Cg_H$ vs. $k_H$. In (**a**), vertical dashed line shows the vertical mean of $N(z)$. In (**d**) and (**e**), $k_H^C$ and $k_H^M$ denote horizontal wavenumbers associated with the cyclone-wave resonance (680 km in wavelength) and the quasi-geostrophic disturbances (100 km). In (**d**), a hatched region shows the range of observed wave frequency. In (**e**), a gray horizontal strip shows the fastest group velocity for $k_H^C$ and $k_H^M$.

Along the vertical profile of $N(z)$ during T/S Oshoro-maru expedition, visiting the position of the FATO mooring, we distinguished the two distinct peaks in the stratification around depths of 30 and 200 m (Fig. 3a). The upper one indicated the base of SML and yielded $N$ = 14 cycle per hour (cph), while the lower one indicated the main pycnocline and yielded $N$ = 6 cph. At depths below the main pycnocline, $N$ fell with depth until reaching nearly 0.2 cph right above the sea floor (~ 1800 m). The baroclinicity of the main pycnocline may play a crucial role in maintaining the geostrophic balance in the top layer above that pycnocline (Fig. 3).

According to the SSH spatial distribution, the main axis of near-surface current showed the prominent meandering feature near the FATO station (bold green in Fig. 1a–c). The maps depicted that the FATO site was located near the southern rim of a cyclonic mesoscale structure in August and September. Near the mooring station, the satellite image describes that the RVA was always positive for the whole summer months because of the cyclonic eddy, with an increase from ${\zeta }_{g}=0.05f$ to $0.2f$ (Fig. S1a).

For the longtime evolution of SSH and RVA horizontal distribution, we discover that the cyclonic eddy rapidly grew up in August and then remained intense throughout the following months of September and October (Fig. S1a–g). Subsequently, the cyclonic feature stayed weak during the winter months, and eventually dissipated by February (Fig. S1h–j).

### Spectral signatures of near-inertial waves along vertical axis

**Figure 4:**
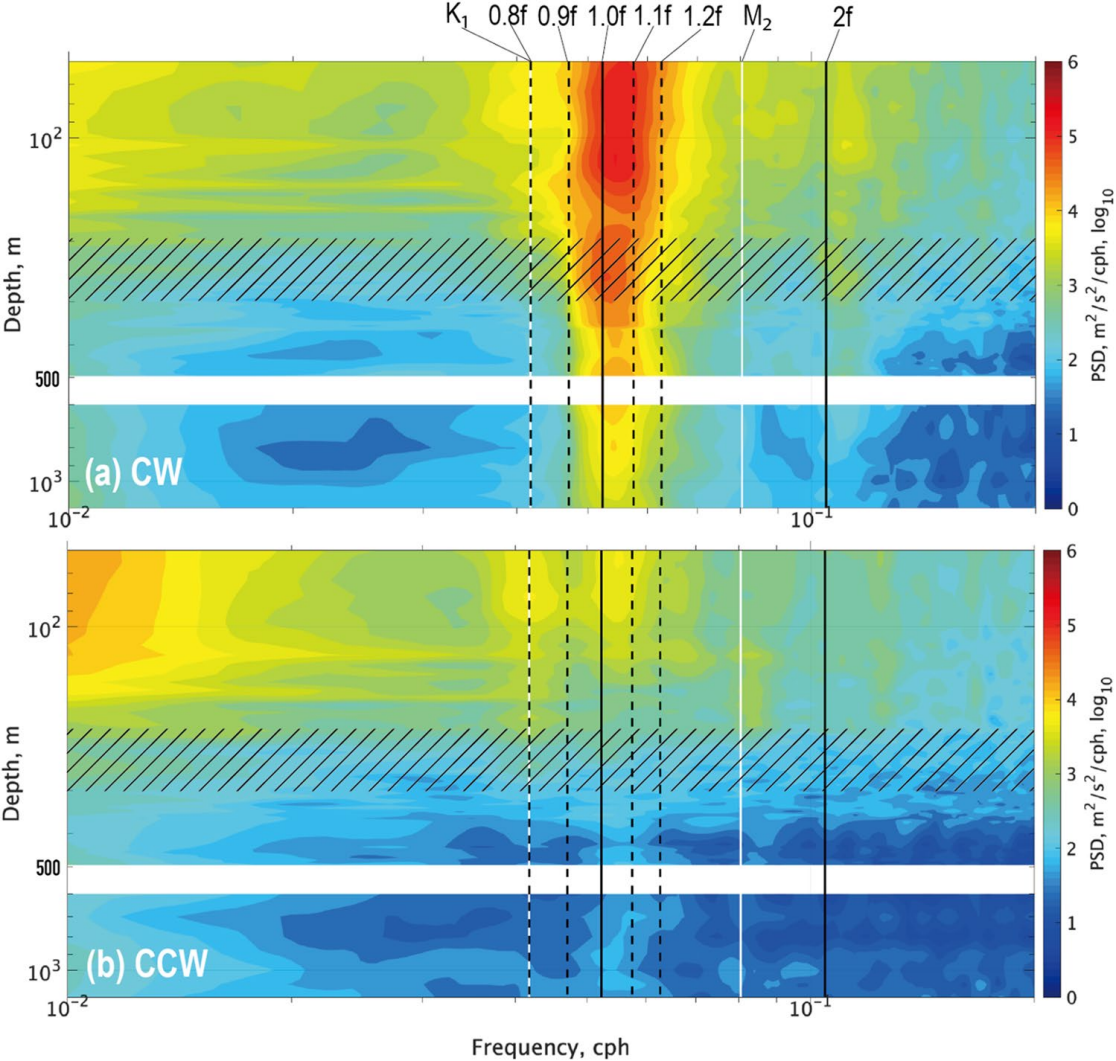
Rotary frequency spectra for the observed horizontal current along vertical axis (Aug. 24–Oct. 31, 2019): (**a**) clockwise (CW) and (**b**) counterclockwise (CCW). Key frequencies are indicated by vertical bars: *f* and 2*f* (black solid); 0.8*f*, 0.9*f*, 1.1*f* and 1.2*f* (black dashed); tidal constituents of K_1_ and M_2_ (white solid). Hatched region shows an approximate range of the main pycnocline (200–300 m depth).

The time-series observations of the horizontal current at the successive depths allowed for the spatial depiction of the vertical distribution of near-inertial oscillation intensity (Fig. 4). In the top layer (200 m deep or shallower), the sub-inertial frequencies of $\omega$ ≤ 10^–2^ cph yielded a profound amount of kinetic energy, especially in the counterclockwise (CCW) rotational direction. This sub-inertial maximum was interpreted as a contribution from the surface-intensified geostrophic current, associated with the TOF jet.

According to the spectral analysis in the clockwise (CW) rotation, we could find a distinct peak around $\omega =f$ across the entire water column, including the bottom layer greater than 1000 m depth (Fig. 4a). In the top layer of the CW component, lying between the SML and the main pycnocline (hatched in Fig. 4), the near-inertial peak appeared to be relatively wide spreading at both sides of $\omega =f$, i.e., 0.9–1.2*f*. When it went toward the deeper section, the peak appeared to be further sharpened, particularly at the sub-inertial side. The waves entering the deeper section were more strictly segregated based on the minimum propagation frequency of ${\omega }_{\text{min}}=f$ due to no ambient flow leaving geostrophic shear significantly as well as the RVA terms in that depth range [Eq. (1)]. The near-inertial peak was found to be less significant in the counterclockwise (CCW) component than in the CW counterpart (Fig. 4b).

The energy contribution in the vicinity of $\omega =f$ (i.e., 0.9–1.2*f*) appeared to mostly result from the modulation locally near the FATO station, rather than from propagating waves from the neighboring latitudes, particularly from north (Fig. 4a). For example, the spectral energy can be found at $\omega =1.1f$, corresponding to the inertial frequency at 42.5°N, which is an unrealistic candidate as a source of typhoon-generated wave KE (Fig. 2a–c).

The Doppler shifting ($\Delta \omega ={\varvec{k}}\cdot {{\varvec{U}}}_{{\varvec{g}}}$) could contribute to the wide-spreading frequencies around $\omega =f$ at the depths above the pycnocline (Fig. 4a). The large-scale waves associated with the cyclone speed can yield the simple estimate of the Doppler shift by $\Delta \omega =\frac{\left|{{\varvec{U}}}_{{\varvec{g}}}\right|}{{V}_{\text{cyc}}}f$. For example, providing the cyclone’s travelling speed ${V}_{\text{cyc}}$ = 8 m s^−1^ (Table 1) and the magnitude of near-surface geostrophic current $\left|{{\varvec{U}}}_{{\varvec{g}}}\right|$ = 0.2–0.5 m s^−1^ would yield a frequency shift of 2–6% from the intrinsic wave frequency.

**Figure 5:**
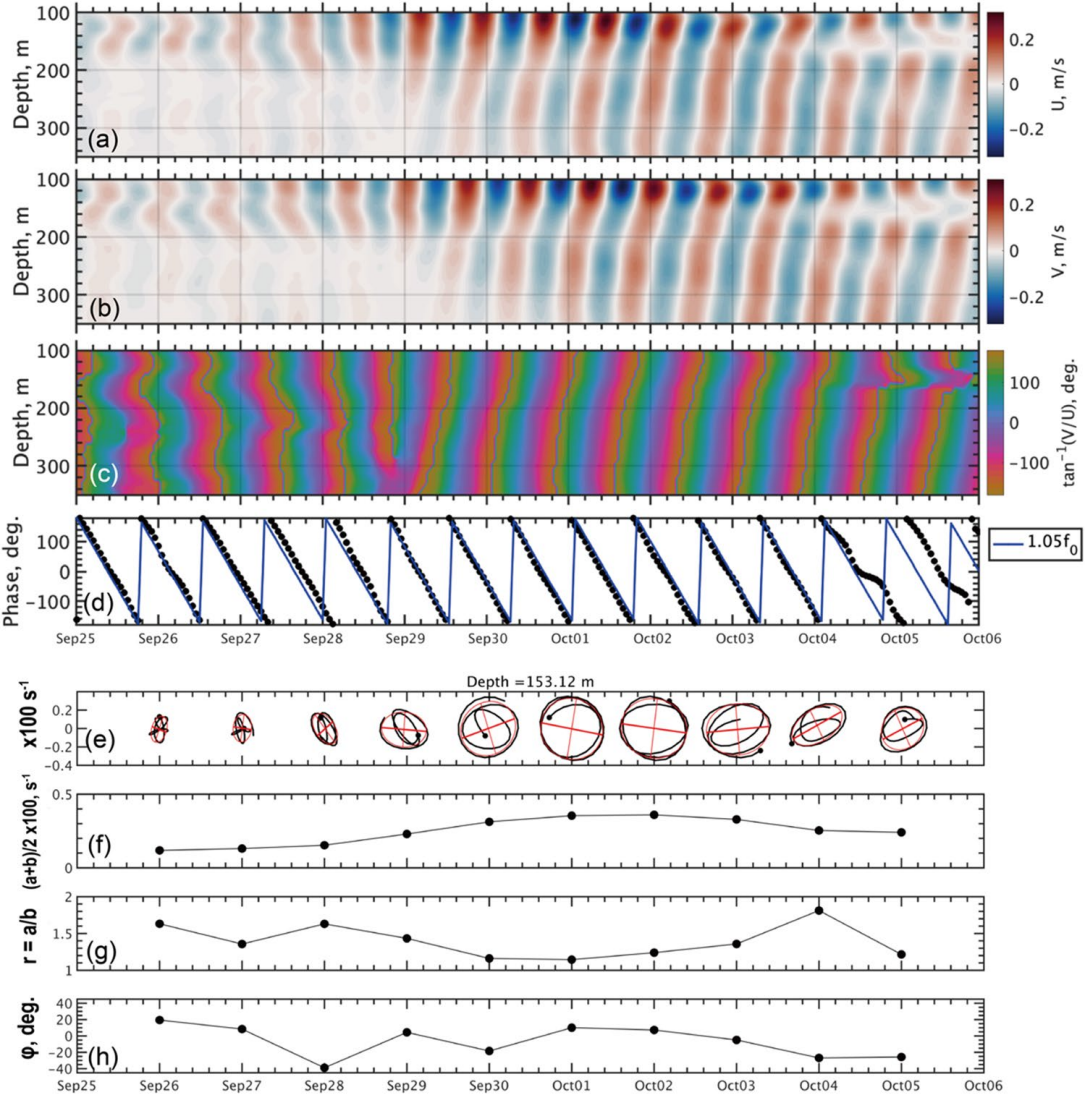
Observed signatures of NIW: (**a**, **b**) near-inertial velocities in zonal and meridional directions, $(U_{NI},V_{NI})$, respectively; (**c**) wave phase $\Phi(t,z)=\mathrm{tan^{-1}}(U_{NI},V_{NI})$; (**d**) $\Phi(t)$ at depth of 153 m shown by black dots. In (**d**), blue lines show the linear regression for the phase evolution deduced from horizontal velocity vectors in (**c**) and expressed by $\Phi(t)=-2{\uppi}{\upomega}(t-t_{0})$, where $\upomega=1.05f$ and $t_{0}$ is the initial time set to 00:00 UTC on September 25. In (**e**), hodographs of vertical shear vector for the near-inertial horizontal current, $(U_z,V_z)$, are shown, for the periods of 48 h, approximately equivalent to 2.5 inertial cycles. Black dots show the initial point. Ellipses in red show the best-fit regressions with the semi-major and semi-minor axes, $a$ and $b$ respectively, which are estimated by the least-square criterion technique. In (**f**–**h**), characteristics of wave properties extracted from the horizontal orbits of the ellipse are shown: (**f**) mean radius $\left(a+b\right)/2$; (**g**) major/minor ratio $r=a/b$; (**h**) tilt angle $\upphi$, with positive values in clockwise.

### Wave properties deduced from oscillatory motions

From September 29 onward, the upward-looking ADCP at the top clearly demonstrated the periodic pattern of near-inertial horizontal velocity propagating in the upward direction over the depths of 100–350 m (Fig. 5a–c). According to the past studies (e.g., Leaman and Sanford^22^), the upward propagation of phase accompanies oppositely directed (downward) propagation for wave kinetic energy. The best-fit regression line based on the phase evolution deduces a frequency of the most-dominant oscillation as $\omega =1.05f$, calculated at a depth of 153 m (Fig. 5d) (c.f., $1.02f$ in the equatorial Pacific^23^).

Vertical shear of near-inertial current commonly depicts a clockwise rotation in hodographs (Fig. 5e). The trajectory was depicted with an hourly near-inertial band-passed velocity, for data chunks of 48 h with a half-overlapping. We also drew best-fit ellipses for the hodographs by using the least-squares criterion technique^24^. When the near-inertial oscillation was the most intense, i.e., between September 29 and October 2, the rotational orbit was little compressed. The ratio between the semi-major and semi-minor axes, respectively $a$ and $b$, was estimated as $r = a/b = 1.1$. Aside from this, the ellipses were only slightly tilted from the horizontal by $\phi =$ 20° or smaller (Fig. 5f,g).

From the fact, the theory of WKB internal waves suggests that the packets of incident waves travelled approximately in the east–west direction^23^. Notwithstanding, we have only little confidence on the consequence because of the modest oblateness for the elliptical regressions (i.e., $r\approx 1$). From the polarization viewpoint, the ratio $r=\frac{a}{b}$ can be related to that of the intrinsic frequency ($\omega$) to the effective Coriolis frequency (${f}_{\text{e}}$)^23^:

2 $$r=\frac{\omega }{{f}_{\text{e}}}$$

Combining it with the dispersion relation:

3 $${\omega }^{2}={{f}_{\text{e}}}^{2}+{N}^{2}\frac{{{k}_{H}}^{2}}{{m}^{2}}$$

where ${k}_{H}=\sqrt{{k}^{2}+{l}^{2}}$ and $(k, l, m)$ are respectively zonal, meridional, and vertical wavenumbers, one can derive an estimate of horizontal wavelength (${\lambda }_{H}=\frac{2\pi }{{k}_{H}}$) for the most dominant NIWs by an equation below:

4 $${\lambda }_{H}=2\pi \sqrt{\frac{{r}^{2}}{{r}^{2}-1}}\left(\frac{N{C}_{z}}{{{f}_{\text{e}}}^{2}}\right)$$

Here, we substituted the relation, ${C}_{z}=\frac{\omega }{m}$, based on the vertical phase translation estimated from the ADCP observations (Fig. 5c). With measured ranges for the important parameters ($r$ = 1.1, $\zeta$ = 0.05–0.20 ${f}_{0}$, $N$ = 4–5 cph, and ${C}_{z}$ = 500–800 m d^−1^), we obtain the estimate of ${\lambda }_{H}$ falling within a typical value of 60–140 km.

### Horizontal wavelength and horizontal group velocity

The surface-delivered NIKE is expected to spatially radiate as internal waves. The horizontal migration is examined based on the rotational internal wave dispersion together with the principle vertical modes, *n*, as follows^25^:

5 $${\omega }_{n}^{2}={f}^{2}+{c}_{n}^{2}{k}_{H}^{2}$$

where ${k}_{H}$ is the horizontal wavenumber; ${c}_{n}$ is the eigenvalue phase speed of the *n*^th^ vertical mode (i.e., *n* = 1, 2, 3, $\cdots$). The dynamic vertical mode calculation was performed using the averaged profile of $N(z)$ during the T/S Oshoro-maru expedition in July (Fig. 3b,c; see “Data and methods”). We evaluated the eigenvalues of ${c}_{n}$: 1.44, 0.62, 0.42, 0.31 and 0.25 m s^−1^ for the first five vertical modes (i.e., *n* = 1, 2, 3, 4, and 5). The wavenumber-frequency relates the intersection of every dispersion curve to the linear lines of the cyclone speed (${V}_{\text{cyc}}$):

6 $${k}_{H}^{C}=2\pi {L}_{n}^{-1}=\frac{f}{\sqrt{{{V}_{\text{cyc}}}^{2}-{{c}_{n}}^{2}}}$$

where ${L}_{n}$ is the wavelength as a function of the vertical mode $n$. Regardless of the mode we have the following approximate relationship: ${k}_{H}^{C}\approx \frac{f}{{V}_{\text{cyc}}}$. Providing ${V}_{\text{cyc}}$ = 10 m s^−1^, the horizontal wavelength ${\lambda }_{H}={L}_{n=1}$ would be approximately estimated at 680 km. The horizontal scale suggested is huge, which can be viewed as the scale of typhoon systems^1,2^.

The derivative of the wave dispersion (5) gives the horizontal group velocity (${Cg}_{H(n)}$) as the vertical mode solution for an arbitrary wavenumber:

7 $${Cg}_{H}=\frac{\partial {\omega }_{n}}{\partial {k}_{H}}=\frac{{c}_{n}^{2}{k}_{H}}{{\omega }_{n}}=\frac{{c}_{n}^{2}{k}_{H}}{\sqrt{{f}^{2}+{c}_{n}^{2}{k}_{H}^{2}}}$$

The analytic solution above illustrates the monotonous positive relationship between ${Cg}_{H}$ and ${k}_{H}$, where it becomes constant to be ${Cg}_{H}={c}_{n}$ in the limit of ${k}_{H}=\infty$. It also suggests a linear increase of ${Cg}_{H(n)}$ with the wavenumber, for the bands of ${k}_{H} \gg {k}_{H}^{M}$, where ${k}_{H}^{M}=\frac{2\pi }{R}=\frac{f}{{c}_{1}}$ with $R$ being a horizontal scale associated with the quasi-geostrophic balanced motions^12,26^. In the present case, $R$ was estimated at roughly 100 km based on the mean profile of $N(z)$ and its first baroclinic eigenvalue (Fig. 3). This is approximately consistent with the horizontal wavelength, 60–140 km, derived from the wave dispersion.

Kawaguchi and Yabe (unpublished) performed the high-resolving numerical simulations for the oceanic reactions to the wind forcing of Typhoon *Tapah*. They showed that the cyclone-resonance scale of ${2\pi /k}_{H}^{C}$ was the most profound irrespective of the presence or absence of the TOF. Also, they showed that the TOF resulted in the quasi-geostrophic disturbances, which empowered the near-inertial internal waves with the similar scale with them (i.e., $R=2\pi {/k}_{H}^{M}$).

Regarding the mesoscale feature, the wave dispersion predicts the frequency at $\omega =$ 1.4*f* for the first baroclinic mode, which is obviously out of the typical range of the observed $\omega$ (Fig. 4). This implies that the first mode cannot exist as the propagating near-inertial waves, so that the second or greater vertical modes may instead determine the fastest travelling speed. Consequently, we estimated ${Cg}_{H}$ $\approx$ 20 km/day for both wavenumbers of ${k}_{H}^{C}$ and ${k}_{H}^{M}$ (Fig. 3e).

It is necessary to clarify the potential influences of near-surface NIKE in remote places from the FATO station (Fig. 2a–c). Overlain with the horizontal distribution of Π, the approximate radii were marked up over which near-inertial waves possibly travelled for a period of three and seven days (Fig. 2a–c). In general, the internal waves of ${k}_{H}^{C}$ and ${k}_{H}^{M}$ are commonly too sluggish to travel far horizontally and can reach ~ 60 km at most for three days. Therefore, it would not be significant at the FATO site immediately unless the typhoon supplies the inertial energy in its neighborhood. Here, we also maintain that the poleward propagation of near-inertial waves should be largely limited by the Coriolis term $f(\theta )$ in Eq. (1) as discussed in the past studies^27^.

### Vertical distribution of wave kinetic energy

**Figure 6:**
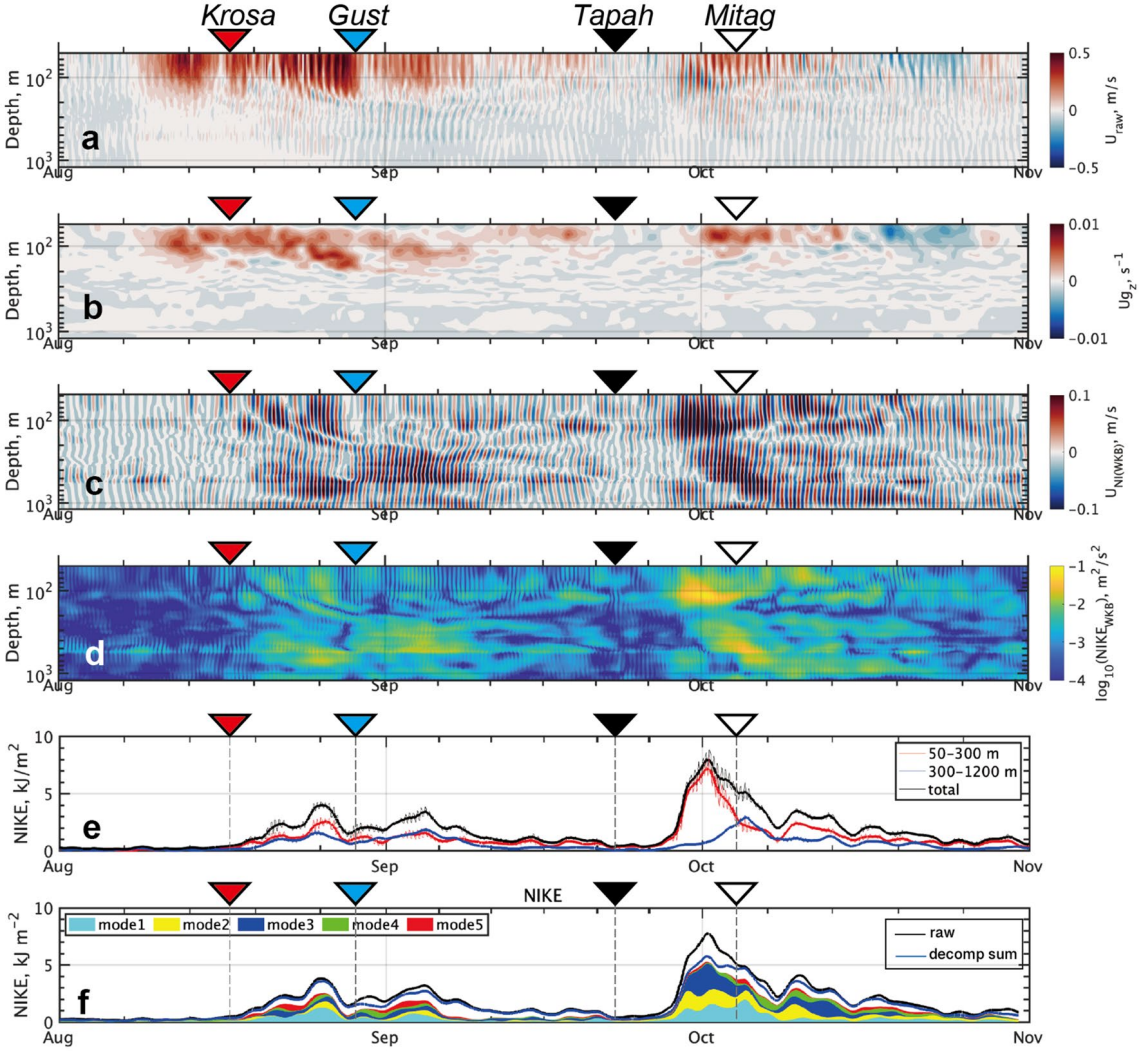
Time-vertical section of eastward component of (**a**) horizontal current, (**b**) low-passed (24 h) vertical shear, (**c**) near-inertial band-passed (0.9–1.1*f*) current and (**d**) its magnitude. In (**e**), the vertical integral of NIKE is shown for depths of 50–300 m (red), 300–1200 m (blue) and the total (black). In (**f**), the contributions from the first five vertical modes are shown by shaded areas, with the total amount accumulating from $n$ = 1 to 20 by bold blue curve, while the raw NIKE by bold black curve. Note that in (**c**–**e**) velocities are WKB-scaled (see “Data and methods” for more details).

The combination of the observed horizontal current at many different depths clearly represented the vertical propagation of NIKE responding to the consecutive passages of the typhoons (Fig. 6). After the near-inertial bandpass filtering and the Wentzel–Kramers–Brillouin (WKB) normalization applied to the horizontal velocity (see the caption of Fig. 6; “Data and methods”), NIKE was dispersively transferred along the time-vertical dimension (Fig. 6c,d). In the late August and the early October, respectively, following *Krosa* and *Tapah*, a part of NIKE was apparently brought down to depths greater than 1000 m. Another part of NIKE appeared to remain in the vicinity of the lower pycnocline (e.g., 300 to 400 m). Following *Tapah*’s passage in between the late September and the early October, there were extremely strong signals of near-inertial oscillations at depths of 100 m or shallower. The intensified NIKE remained at a similar depth for a few weeks and did not show any downward propagation until it dissipated eventually.

We also calculated the modal decomposition for near-inertial band-passed horizontal velocity (Fig. 6f). Please refer to “Data and methods” and Fig. S2 for more details. The decomposition clarifies the allocation of the observed NIKE by each vertical mode. In general, the first five vertical modes show the dominant contribution for the profound events in NIKE following typhoon passages, covering roughly 60–90% of the total amount. Regarding the events in August and early September, the first vertical mode explains roughly 35% of all, while the second and third modes do approximately 15 and 10%, respectively. During the greatest NIKE elevation observed following *Tapah*, the third mode claims the relatively large contribution by more than 40%, while the first and second modes share the remainder parts of 20–30%.

To quantitatively discuss its vertical allocation, the wave-related NIKE was accumulated and compared between the two vertical segments of the top layer (50–300 m) and the bottom layer (300–1200 m), separated by the main pycnocline (Fig. 6e). For the estimate, we integrated the WKB-scaled NIKE over the ${N}_{0}$-standardized vertical coordinate ${z}^{*}$ (see “Data and methods”). Overall, both cumulative curves displayed the similar responses to the cyclone passages. Commonly, the greatest peaks for the two segments lagged by about a week, relative to the surface energy input. The sum of NIKE was roughly comparable between the two segments. The peaks coincidental between the two layers suggested that a major part of the amount of wave kinetic energy was transported through the bottom by the near-inertial wave packet shortly after the cyclone’s passage.

A notable exception of the near-inertial wave event was found to occur around October 1 (Fig. 6e). During this event, the maximum energy in the top layer, most likely as its response to *Tapah*, preceded the response of the bottom layer even by several days. Also, its integrated amount over the top layer reached 7 kJ m^−2^ or greater, which surpassed by far the response of the bottom layer (e.g., more than double). The maximum wave kinetic energy shortly attenuated to half or smaller in 10 days (Fig. 6e).

Following *Tapah*, the slab model estimated the gross amount of the surface NIKE input at around 6.5 kJ m^−2^ (Fig. 2h). Observed wave NIKE integrated over the top layer was comparable with the value estimated from the slab model. As the cause of excess NIKE of the top layer, we hypothesize the possibility of the temporary expansion of wave kinetic energy due to the rapid change in vertical group velocity. In the next section, we explore this hypothesis from the Lagrangian viewpoint for the near-inertial wave packet in the vertical direction.

As already mentioned above, NIKE in the upper part of water column showed a 5–6 days delay after the passage of *Tapah* (Fig. 6c–e) despite the slab model that simulates the immediate response to it in the SML (Fig. 2f). We hypothesize that a combination of potential factors caused the delayed response of NIWs and its noticeable amplitude. First, we focus on the sluggish geostrophic current near the surface that lasted for the period of September 21–28, which included the *Tapah*’s passage (Fig. 6a). The sluggish current is likely due to the relative location of a cyclonic eddy (CE). We infer that the core of CE lied right over the station, resulting in such a weak geostrophic current observed. We suppose that the CE, characterized by a positive RVA, could block the NIW packets entering inside the structure, consistent with the numerous previous studies^5,11,18,29^. During the shipboard survey near the FATO station in the late October, 2019, we indeed observed apparently weak signals of NIKE within the CE mesoscale structure compared to those in the ambient waters (Fig. 6 of Kawaguchi et al.^18^).

Second, we suggest a possible scenario that the delayed peak of NIKE was attributable for the late arrival of energy-containing NIW packets emanating from remote source regions. In fact, we spot the potential candidate as an abundant NIKE source, centered around (40°N, 136°E), at the distance of 5-day from the FATO station assuming the traveling speed of 20 km day^−1^ (Fig. 2b). For the third point, we know that vertical shear of the near-surface mean current concomitantly appeared along with the rapid growth of NIKE (Fig. 6b,c). From the fact, it is plausible that the mean current’s vertical shear promoted the amplification of the NIW. This process will be discussed in the following section.

## Discussion

**Figure 7:**
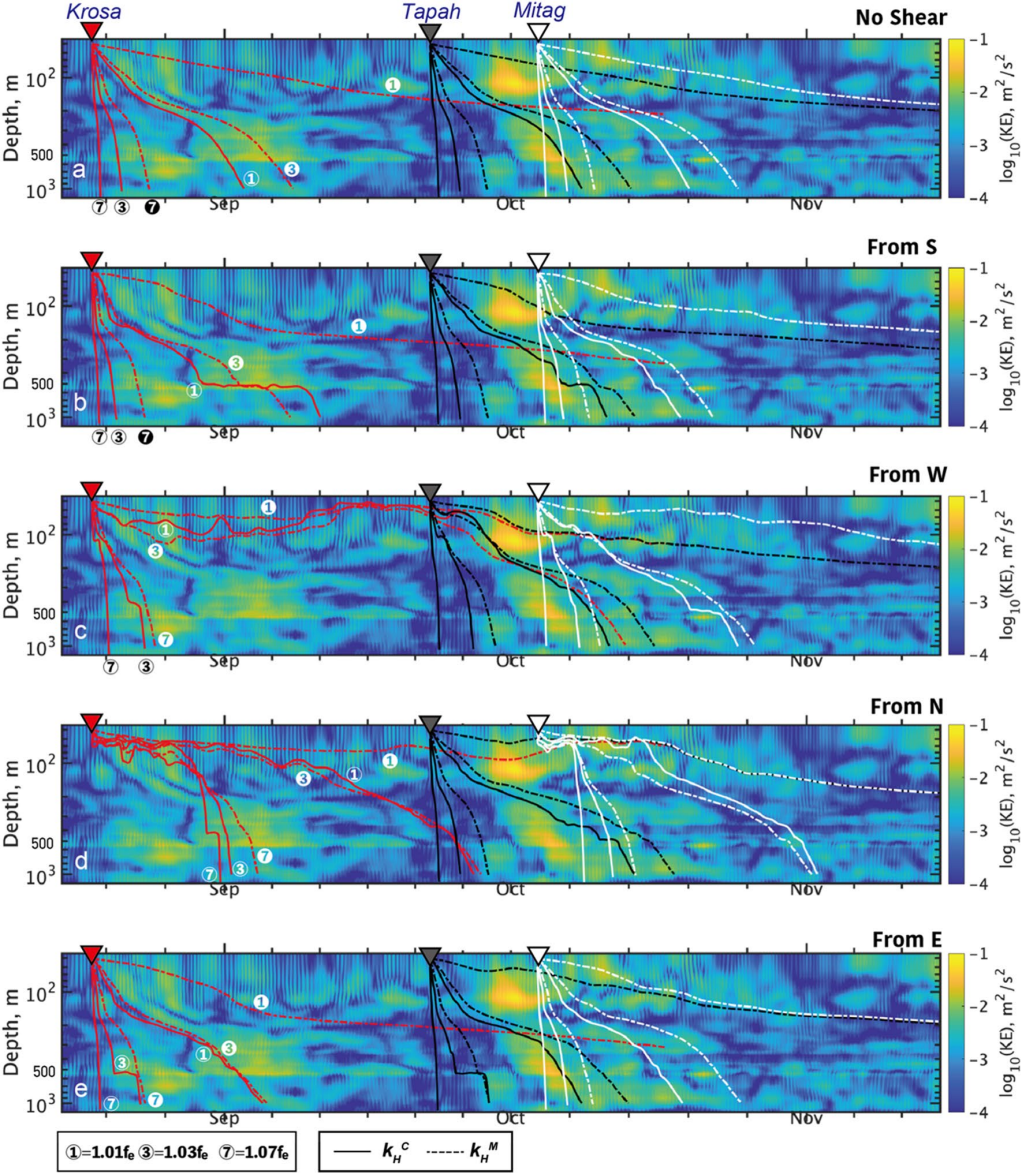
Wave rays predicted by the buoyancy term of Eq. (8) overlain with near-inertial kinetic energy in the background shading. Curves in red, black, and white show those generated by *Krosa*, *Tapah*, and *Mitag*, respectively. The selected paths are examined for frequencies of $\omega=1.01f_e,\boldsymbol{ }1.03f_e,\boldsymbol{ }\mathrm{and}\; 1.07f_e$, while horizontal wavenumbers of $k_H=k_H^M$ and $k_H=k_H^C$ shown by dashed and solid curves, respectively. As an example, numbers of $\omega$ and $k_H$ (subscript) are shown for the curves of *Krosa*. Note that wave kinetic energy in background is WKB-scaled.

We based our discussion on examining the paths of wave propagation, pursuing how it behaved depending on the characteristics of incident waves in response to the vertical group velocity (${Cg}_{z}$) as follows^11^:

8 $${Cg}_{z}=-\frac{{\left({\omega }^{2}-{{f}_{e}}^{2}\right)}^{3/2}}{fN{k}_{H}}-\frac{{\omega }^{2}-{{f}_{e}}^{2}}{{{N}^{2}{k}_{H}}^{2}} \bigg(\frac{\partial {U}_{g}}{\partial z}l-\frac{\partial {V}_{g}}{\partial z}k \bigg)$$

where the first term represents the vertical structure of density stratification $N(z)$; the second and third terms are the cross product between vertical shear of geostrophic current $\frac{\partial {{\varvec{U}}}_{{\varvec{g}}}}{\partial z}=\left(\frac{\partial {U}_{g}}{\partial z},\frac{\partial {V}_{g}}{\partial z}\right)$ and wavenumber vector ${\varvec{k}}=(k,l,m)$, respectively. The wave packets are initially situated at the times of the three typhoon events (Table 1) and at a depth of 50 m, with the initial conditions of $\left(\omega , {k}_{H}\right)$ and vertical shear of geostrophic ambient flow ${(U}_{g},{V}_{g})$ (Fig. 6b). The computation ended either at the maximum time of 60 d or when the particle arrived at a depth of 1000 m. In the ray-tracing experiments, the six different conditions of $(\omega , {k}_{H})=\left(1.01{f}_{e},{k}_{H}^{C}\right)$, $\left(1.03{f}_{e},{k}_{H}^{C}\right)$, $\left(1.07{f}_{e},{k}_{H}^{C}\right)$, $\left(1.01{f}_{e},{k}_{H}^{M}\right)$, $\left(1.03{f}_{e},{k}_{H}^{M}\right)$, and $\left(1.07{f}_{e},{k}_{H}^{M}\right)$ were explicitly investigated (Fig. 7) (see “Data and methods” for further information).

### Dependencies of wave rays on ${k}_{H}$ and $\omega$

In the default experiment, the shear term in Eq. (8) (see “Data and methods”) was not included. Principally, the vertical distribution of $N$ determines the common behaviors of the wave ray––the greatest descending speed can be viewed in the top layer, underneath the SML, followed by the significant attenuation near the main pycnocline at a depth of around 300 m (Fig. 7a). Then, the descending speed recovered in the deeper section underneath the pycnocline continuing through the bottom of the data record, 1200 m.

As was mentioned already, the horizontal wavenumber dominantly determined the magnitude of ${Cg}_{z}$ (Fig. 7a). The cyclone-internal wave resonant scale (${k}_{H}={k}_{H}^{C}$) showed the greatest descending speed, typically travelling 1000 m within 5 d for any choice of $\omega$. Meanwhile, the smaller scales of near-inertial waves (${k}_{H}={k}_{H}^{M}$) generally showed sluggish downward energy propagation, resulting in the absolute postponement of the wave kinetic energy delivery to the deeper section. For the case of $\omega =1.07{f}_{e}$, the mesoscale would take roughly 3 weeks or more. Accordingly, a variety of wave rays emanating from the consecutive typhoons of *Tapah* and *Mitag* intersected with each other, creating the intricate distribution of oscillatory wave kinetic energy across a major part of the water column.

In terms of the frequency of incident waves, it provided the modification in the descending speed of the wave packet (Fig. 7). Qualitatively, high $\omega$ tended to rapidly descend to get to the bottom, as opposed to low $\omega$. Quantitatively, the wave packet of $\omega =1.07{f}_{e}$ arrived at 1200 m within 5–6 days, while $\omega =1.01{f}_{e}$ took at least 25 d. In fact, the observed distribution of NIKE was in close agreement with some of the predicted curves based on the first term of Eq. (8) (Fig. 7a). For example, following the passage of *Krosa*, the three distinctive wave rays were clearly visible. We can discover the guidance by the wave variants of ($\omega$, ${k}_{H}$) $=(1.03{f}_{e}$, ${k}_{H}^{M}$) descending to 900 m depth arriving around August 23, while $(1.01{f}_{e}$, ${k}_{H}^{C}$) and $(1.03{f}_{e}$, ${k}_{H}^{M}$) to 600 m depth around September 1 and 6, respectively.

As for *Tapah*, the variants of $(1.01{f}_{e}$,${k}_{H}^{C}$) and $(1.03{f}_{e}$, ${k}_{H}^{M}$) were only discernable, which may guide the energy down through the bottom of the current observation, 1200 m. For *Mitag*, the observed NIKE distribution was totally disturbed presumably due to the intersection of numerous rays from the consecutive typhoons of *Tapah* and *Mitag*. From the complicated NIKE distribution, any ray with significant amplitude was not discernible at the bottom layer beyond the main pycnocline.

### Ray’s refraction due to baroclinicity

**Figure 8:**
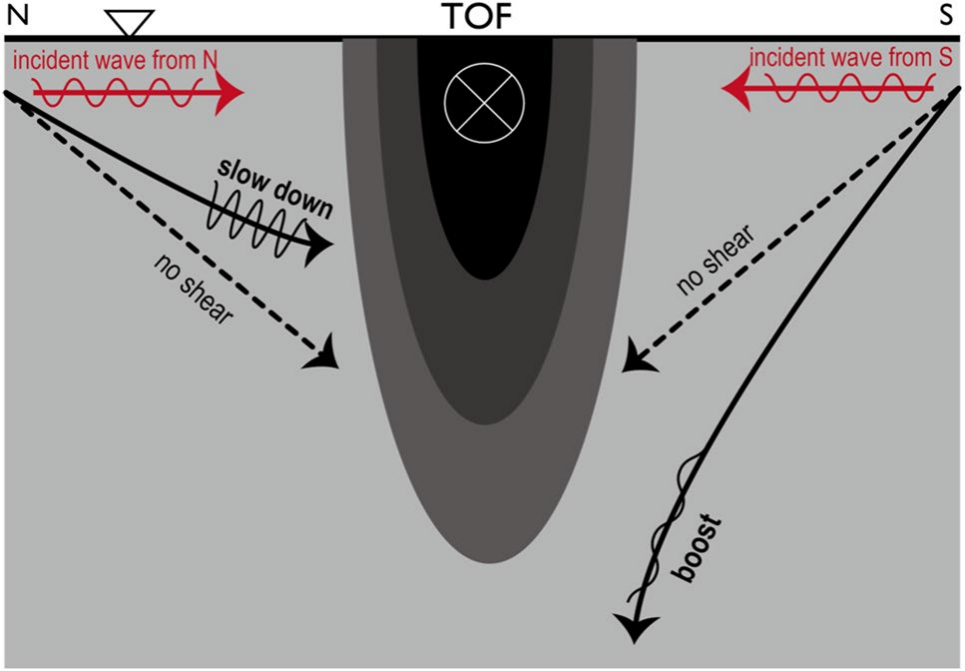
Simplified schematic of ray refraction of near-inertial waves, associated with the vertical shear of TOF. The TOF current is assumed to be surface-intensified and go towards due east. When incident waves come from north as approaching the TOF, the ray cannot descend compared to the depth determined by the buoyancy term [Eq. (8)]. When waves come from south, they can go deeper than that. Please refer to “Data and methods” for further details.

Vertical shear of the baroclinic current provides a significant modification in the travelling path of the waves (Eq. (8)). Its intensity of the cross product ($\frac{\partial {{\varvec{U}}}_{{\varvec{g}}}}{\partial z}\times {\varvec{k}}$) is maximized when an incident wavevector orthogonally meets the baroclinic flow (see the illustration in Fig. 8). Throughout summer and fall in 2019, the main TOF axis was oriented in the northeast or occasionally southeast directions (Fig. 1a–c). Vertical shear, ($\frac{\partial {U}_{g}}{\partial z}, \frac{\partial {V}_{g}}{\partial z}$), was horizontally in a similar direction with the surface geostrophic current since it is mostly surface intensified (Fig. 6a,b). The maximum magnitude of vertical shear was found around depths of 70–200 m. Baroclinic shear was generally noticeably profound in strength between the early August and the mid October as altering the primarily contributing components. The period of the maximum shear was fully overlapped with the recurrent typhoon passages (Table 1).

We examined the trajectories of the incoming waves emanating from the different source regions (south, west, north, and east) relative to the site (Fig. 7b–e). For *Krosa*, which delivered the greatest energy in the west and southwest (Fig. 2a), the mesoscale waves (e.g., 100 km) were significantly modulated to slow down as a result of vertical shear enhanced in the late September (Fig. 6c,d). Some rays from *Krosa* were persistent in the early October even after *Tapah* passed.

The rays from *Tapah* were relatively less modified than those from the others by the buoyancy term due to weakened vertical shear throughout October (Fig. 7). Exceptionally, the rays from north, in particular, with the mesoscale wavelengths showed the noticeable refraction beyond the buoyancy term. The downward-going waves appeared to bounce back toward the surface, coincident with the maximum of observed NIKE (Fig. 6e), and remained stagnant, overlapped with NIKE of the new-coming *Mitag*. Rays from *Mitag* were relatively less refracted by the shear term for any direction except for north (Fig. 2c). We maintain that near-inertial waves emanating from the south regions relative to the site is unlikely to be significant in amplitude due to the restriction of ${\omega }_{\text{min}}(\theta )$ for the northward propagation [i.e., Eq. (1)].

## Concluding remarks

According to the mixed-layer slab model simulations, we depicted the horizontal distribution of surface inertial energy. During the passage of *Krosa*, the significant energy patch was positioned in the southwest direction about several days’ distance from the mesoscale wavelengths (i.e., $R$) (Fig. 2a). In our hypothesis, the southward component of the horizontal wave propagation could slow down the downward energy propagations. *Tapah* delivered the significant amount of NIKE within the accessible range, in particular, in the northern part of the FATO station. In particular, the mesoscale or smaller-scale wavelengths are more likely subject to the shear effect due to the baroclinicity, resulting in the modification of ${Cg}_{z}$. In contrast, the cyclone-resonant scale is less likely subject to the effect because it immediately descends to the bottom layer before interacting with the front.

Considering the conservation law of the wave action ($\mathcal{A}=\frac{E}{\omega }$, where $E$ is the wave kinetic energy) with respect to the vertical direction, the rapid change of ${Cg}_{z}$, resulting in the convergence, must be complemented by an increase of the wave amplitude^7^, i.e.:

9 $$\frac{D\mathcal{A}}{Dt}=-\mathcal{A}\nabla \cdot Cg\approx -\mathcal{A}\frac{\partial \left({Cg}_{z}\right)}{\partial z}$$

We here note that $\omega$ does not change along the trajectory^28^. This implies that the decelerated group velocity, associated with geostrophic shear, may result in the significant amplification of near-inertial waves. The phenomena based on the invariant action flux is also confirmed for near-inertial waves arrested within a negative RVA body^15,29,30^. In fact, near-inertial waves reacting with *Tapah* displayed the remarkable enhancement in the oscillation amplitude at a shallow depth of around 100 m (Fig. 6d). We suppose that the incident wave packet emanating from the northwestern energy source was enhanced through the amplification mechanism, as was mentioned above.

For the future perspectives, more thorough and more accurate estimate for the kinetic energy distribution associated with typhoon passages will require performing the in-situ observations in wider regions across the entire Sea of Japan.

## Data and methods

The FATO mooring station was situated at the geographical point (38.72°N, 137.83°E), roughly 10 km northwest relative to the Sado Island (a mark in Fig. 1a–c)^15,18^. The full water depth at the site is 1770 m.

### Current measurements

In the present study, we mainly analyzed the horizontal current velocities for the period of July 1 and October 31, 2019. To track the vertical near-inertial wave propagation, we deployed a total of nine sets of current meters along the mooring system (Fig. 1g): 75 kHz and 300 kHz WH ADCPs (RD Instrument, US); three Infinity EMs (JFE Advantech Ltd., Japan); four 2 MHz AquaDopp 6000 m (Nortek, Norway) at respective nominal depths of 390, 420, 600, 700, 800, 900, 1000, 1100, and 1200 m. The 75 kHz and 300 kHz WH ADCPs were vertically oriented upward and downward, respectively. The top and bottom WH ADCPs measured oceanic currents at 40 levels over a vertical range of 57–369 m and eight levels over 438–494 m, respectively, with a constant vertical interval of 8 m. Temporal interval was constantly 1 h for every current meter.

The recorded current data were post-processed with a median filter for the removal of outliers. The estimated magnetic declination of 8.9 degrees in October 2019 (the World Magnetic Model) at the mooring location was corrected before the main analyses. The precision of horizontal velocity is 0.005 m s^−1^ for the RD and Nortek instruments and 0.01 m s^−1^ for the JFE Advantech instruments.

### Hydrographic observations

During the shipboard observation of T/S Oshoro-Maru (Hokkaido University), we visited the FATO mooring station (more precisely at position within 1 nautical mile) on July 20 to take a couple of full-depth vertical profiles of conductivity-temperature-depth (CTD) variables by using an SBE-9plus (Seabird Electronics, Inc., US). The raw CTD profiles were averaged into a 1 dbar vertical pitch. The salinity was regularly calibrated with the high precision salinometer, AUTOSAL-8400B (Guildline Instruments, Canada) with the bottle sampled waters. The mean profile of $N(z)$ is created as an average of the full-depth CTD profiling (Fig. 3a).

### Surface geostrophic current from satellite altimetry

For the detection of mesoscale features near the FATO station, we used the satellite-based altimeter dataset distributed by the Copernicus Marine Environment Monitoring Service (CMES) (Fig. 1a–c). The sea surface height (SSH) and SSH-based geostrophic velocities were calculated during the multiple satellite investigations (e.g., TOPEX-Poseidon and Jason 1–3). The horizontal resolution was 1/4 degree (roughly 20 km). Temporal interval was daily. The geostrophically balanced RVA, ${\zeta }_{g}$, was computed at every grid cell, using the midpoint differential method^10^.

### Mixed layer slab model

For the source regions for the near-inertial wave packets by the typhoon events, the delivered wave kinetic energy input at the surface layer was calculated on the geographical map across the Sea of Japan. For this computation, we used the mixed layer slab model^3,31^. In the slab model, the kinetic energy of inertial motion in the SML is numerically solved thus:

10 $$\frac{1}{2}\frac{d{Z}_{I}^{2}}{dt}=-r{\left|{Z}_{I}\right|}^{2}-R\left[\frac{{Z}_{I}}{{D}^{*}H}\frac{d{T}^{*}}{dt}\right]$$

where ${Z}_{I}={U}_{ML}+i{V}_{ML}$ is the inertial component of horizontal velocity; $\rho T={C}_{D}{\rho }_{a}{{\varvec{U}}}_{{\varvec{a}}}\left|{{\varvec{U}}}_{{\varvec{a}}}\right|$ is the surface wind stress, where ${{\varvec{U}}}_{{\varvec{a}}}=$ ${U}_{a}+{iV}_{a}$ is the 10-m height wind velocity in the complex form, and ${\rho }_{a}$ = 1.2 kg m^−3^ the air density. In the present study, we set the drag coefficient that is variable and dependent on the 10-m wind speed $\left|{{\varvec{U}}}_{{\varvec{a}}}\right|$^32^:

11 $${C}_{D}=\left\{\begin{array}{ll}1.3 \times {10}^{-3} & \text{if }\left|{{\varvec{U}}}_{{\varvec{a}}}\right|<10 \mathrm{m\space} {\mathrm{s}}^{-1}\\ \left(0.49+0.065|{\varvec{U_{a}}}|\right)\times {10}^{-3} & \text{ if }\left|{{\varvec{U}}}_{{\varvec{a}}}\right|\ge 10 \mathrm{ m\space}{\mathrm{s}}^{-1}\end{array}\right.$$

The wind-dependent coefficient yields the wind stress 29% and 57% greater for the cases of $\left|{{\varvec{U}}}_{{\varvec{a}}}\right|=$ 15 and 20 m s^−1^ if compared to those with the fixed value of ${C}_{D}=1.3 \times {10}^{-3}$. We also add that the drag coefficient chosen here was widely used for the studies that quantify the impacts of atmospheric disturbances to the ocean^33,34^.

$H$ is the mean thickness of SML, and we set it to be 20 m according to the hydrographic observation at the site (Fig. 3a). The superscript (*) signifies the conjugate of complex numbers, namely, ${D}^{*}=d-if$ and ${T}^{*}=\frac{{\tau }_{x}-{i\tau }_{y}}{\rho }$, where $d$ is the damping constant for the inertial motion of the SML. We assumed in the calculation that ${d}^{-1}$ = 4 day. The choice of $d$ potentially brings some differences in the results. We evaluated its uncertainty by comparing the results for ${d}^{-1}$ = 3–5 day and found a ~ 7% difference in the accumulated net NIKE flux at the surface (Fig. 2h)^35^.

For the calculation of the surface wind stress, the surface wind velocity was retrieved from the distributed dataset of “grid point value of mesoscale model (GPV-MSM)” published by the Japan Meteorological Agency (JMA) (Fig. 1d–f). The horizontal resolution was 1/20° in latitude and 1/16° in longitude. The temporal interval was hourly.

We stress that the amount of wave kinetic energy supplied by a travelling typhoon is not necessarily dependent of its intensity. Alternatively, its travelling speed can be another important factor to determine the oscillation strength. In other words, a slow-moving cyclone may be subject to the cancellation by the preexisting counterflow in the same layer. The fast-moving cyclone has a reduced chance of the cancellation.

### Ray-tracing experiments

For an understanding of the observed energy allocation, it provides a merit to track the pathways of near-inertial wave migration in the vertical sense. We conducted the ray-tracing investigation based on the simplified dispersion relationship of near-inertial waves^11^. Along the vertical axis, the location of a certain wave packet was tracked by integrating the vertical group velocity in time thus:

12 $$z(t)=\int {Cg}_{z}dz$$

In the continuously stratified water column, we assume^11^:

13 $${Cg}_{z}=\frac{\partial \omega }{\partial m}\approx -\frac{{N}^{2}{{k}_{H}}^{2}}{f{m}^{3}}-\frac{1}{{m}^{2}}\bigg(\frac{\partial {U}_{g}}{\partial z}l-\frac{\partial {V}_{g}}{\partial z}k \bigg)$$

where ${\varvec{k}}=(k,l,m)$ is the wavenumber vector; and ${k}_{H}=\sqrt{{k}^{2}+{l}^{2}}$ is the magnitude of the horizontal wavenumber components. For the simplicity, the vertical wavenumber was determined using the following simplified version of the wave dispersion^36^:

14 $${m}^{2}=\frac{{N}^{2}{{k}_{H}}^{2}}{{\omega }^{2}-{{f}_{e}}^{2}}$$

For the range of $\omega$ = 1.01–1.07*f*, one would obtain the rough estimates of 1500–4000 m for the cyclone-resonant scale of 680 km ($=2\pi /{k}_{H}^{C}$), while 250–650 m for the mesoscale disturbances of 100 km ($=2\pi /{k}_{H}^{M}$). In the calculation above, we used the canonical value of $N={N}_{0}=$ 3 cph^37^. Dividing the full depth of ~ 1800 m by the estimated vertical wavelength would give a simple estimate of the vertical mode; ${k}_{H}^{C}$ corresponds to $n = 1$, while ${k}_{H}^{M}$ does the higher modes of $n$ ≥ 3.

Equation (13) was re-arranged as follows:

15 $${Cg}_{z}=-\frac{{\left({\omega }^{2}-{{f}_{e}}^{2}\right)}^{3/2}}{fN{k}_{H}}-\frac{{\omega }^{2}-{{f}_{e}}^{2}}{{{N}^{2}{k}_{H}}^{2}} \bigg(\frac{\partial {U}_{g}}{\partial z}l-\frac{\partial {V}_{g}}{\partial z}k \bigg)$$

The above relationship describes the dependency of ${Cg}_{z}$ on the horizontal scale of propagating waves––with a large-scale internal wave, the descending rate of a wave packet tends to increase, as opposed to a small-scale internal wave. The frequency $\omega$ and horizontal wavenumber ${k}_{H}$ are explicitly given to examine the influences on the results.

In this study, we considered the frequencies of $\omega =$ $1.01{f}_{e}, 1.03{f}_{e},$ and $1.07{f}_{e}$ as the feasible range of the propagating waves. For the horizontal wavenumber, we tested the following options of ${k}_{H}^{C}$ = 9 × 10^–6^ rad m^−1^ and ${k}_{H}^{M}$ = 6 × 10^–5^ rad m^−1^, respectively, representing the scales associated with the cyclone-induced resonance and the mesoscale features. For $N(z)$, we used the 1-m average from the onsite expedition in July (Fig. 3a). Vertical shear of geostrophic current, ($\frac{\partial {U}_{g}}{\partial z}, \frac{\partial {V}_{g}}{\partial z}$), was obtained as the vertical gradient of the 24-h low-passed horizontal velocity (see Fig. 6b). Subsequently, it was interpolated to the locations of $N(z)$ data points along the vertical coordinate.

In Eq. (13), the first term mostly represents the vertical structure of the density stratification, referred to as the buoyancy term. Meanwhile, the second term represents vertical shear of the geostrophic current, referred to as the shear term, with a vertical component of the cross product with the horizontal wavenumber vector ${\varvec{k}}$, namely,

16 $$\frac{\partial {{\varvec{U}}}_{{\varvec{g}}}}{\partial z}\times {\varvec{k}}=\left(\begin{array}{c}\frac{\partial {U}_{g}}{\partial z}m\\ -\frac{\partial {V}_{g}}{\partial z}m\\ \frac{\partial {U}_{g}}{\partial z}l-\frac{\partial {V}_{g}}{\partial z}k\end{array}\right)$$

We considered some realistic situations that an internal wave packet approached the geostrophic jet that flowed in parallel to the TOF (Fig. 8). On the horizontal plane, the directions of the phase and group velocities were identical. If a wave packet comes from the left side relative to the jet (i.e., $\frac{\partial U}{\partial z}l-\frac{\partial V}{\partial z}k<0$) the downward migration of the packet slows down or is flipped in the principal direction toward the surface (Fig. 8). If the wave comes from the right ($\frac{\partial U}{\partial z}l-\frac{\partial V}{\partial z}k>0$), it boosts the downward speed of the wave packet migration.

### Application of WKB scaling

For the fair representation of near-inertial wave amplitude, the near-inertial band-passed horizontal velocities ${{\varvec{U}}}_{{\varvec{N}}{\varvec{I}}}=({U}_{NI}, {V}_{NI})$ were rescaled based on the WKB theory^24^ by using the observed vertical profile of $N(z)$ thus (Fig. 3a):

17 $${{{\varvec{U}}}_{{\varvec{N}}{\varvec{I}}}}^{\boldsymbol{*}}={{\varvec{U}}}_{{\varvec{N}}{\varvec{I}}}/{\left(N(z)/{N}_{0}\right)}^{1/2}$$

where ${N}_{0}$ = 3 cph representing the canonical intensity of the density stratification^37^. For the integrating procedure of the WKB-scaled wave kinetic energy, the vertical coordinate was stretched by:

18 $${dz}^{*}=\left[N(z)/{N}_{0}\right]dz$$

The new vertical coordinate yields the maximum depth of about 750 m (c.f., 1200 m in the original coordinate).

### Modal decomposition

The modal decomposition of the observed horizontal velocity is performed with the least-squared technique based on the eigenfunctions derived from the in-situ profile of $N(z)$^38^ (Fig. 6f). For the vertical profile, we used the mean profile obtained during the Oshoro-maru cruise in July (Fig. 3a). The arrays of the observed near-inertial band-passed current (Fig. 6c) are linearly interpolated and extrapolated onto the uniformly spaced vertical coordinate at the 1-m interval, between the depths of 0 and 1200 m, corresponding to the original CTD profile.

The modal solutions describe the zonal motion of seawater for each vertical mode, $n$, at an arbitrary depth:

19 $$u\left(z,t\right)=\sum_{n=0}^{\infty }{u}_{n}\left(z,t\right)=\sum_{n=0}^{\infty }\left[{U}_{n}\left(t\right)\frac{\partial {\phi }_{n}\left(z\right)}{\partial z}\right]$$

Regarding the meridional velocity, $v$, it is defined and solved in the similar way with $u$. Here, we assume the WKB-type solutions (Fig. 3b,c) expressed by:

$${\phi }_{n}=\frac{{\left(-1\right)}^{n}}{n}\sqrt{\frac{\overline{N}}{N}}\mathrm{sin }\left(\frac{n\pi }{\overline{N}h}{\int }_{0}^{z}N\left({z}^{^{\prime}}\right)d{z}^{^{\prime}}\right)$$

20 $$\frac{\partial {\phi }_{n}}{\partial z}=\frac{{(-1)}^{n}}{h}\pi \sqrt{\frac{N}{\overline{N}}}\mathrm{cos }\left(\frac{n\pi }{\overline{N}h}{\int }_{0}^{z}N\left({z}^{^{\prime}}\right)d{z}^{^{\prime}}\right)$$

where $h$ is the bottom depth. The equation assures the boundary condition of ${\phi }_{n}=0$ at $z = 0$ and $z =-h$. In the present study, we applied the boundary condition at the greatest observational depth of 1200 m. The average value of $\overline{N }$ is given by

21 $$\overline{N }=\frac{1}{h}{\int }_{-h}^{0}N(z)dz$$

Using the normal modes and the near-inertial band-passed horizontal velocity from the mooring observation, (${U}_{n}, {V}_{n}$) are estimated by solving the following matrix equations:

22 $${\varvec{A}}={{\varvec{\Phi}}}^{-1}{\varvec{B}}$$

where the vectors, ${\varvec{A}}$**, B,** and ${\varvec{\Phi}}$, are given by

$${\varvec{A}}=\left({U}_{0} {U}_{1} \cdots { U}_{M}\right)$$

23 $${\varvec{B}}=\left(\begin{array}{c}\begin{array}{c}\sum_{{\varvec{j}}=1}^{{\varvec{J}}}u\left(j\right)\dot{{\phi }_{0}}\\ \sum_{{\varvec{j}}=1}^{{\varvec{J}}}u\left(j\right)\dot{{\phi }_{1}}\\ \vdots \end{array}\\ \sum_{{\varvec{j}}=1}^{{\varvec{J}}}u\left(j\right)\dot{{\phi }_{M}}\end{array}\right)$$

$${\varvec{\Phi}}=\left(\begin{array}{ccc}\sum_{{\varvec{j}}=1}^{{\varvec{J}}}\dot{{\phi }_{0}}\dot{{\phi }_{0}}& \boldsymbol{ }\cdots & \sum_{{\varvec{j}}=1}^{{\varvec{J}}}\dot{{\phi }_{0}}\dot{{\phi }_{M}}\\ \vdots & \ddots & \vdots \\ \sum_{{\varvec{j}}=1}^{{\varvec{J}}}\dot{{\phi }_{M}}\dot{{\phi }_{0}}& \cdots & \sum_{{\varvec{j}}=1}^{{\varvec{J}}}\dot{{\phi }_{M}}\dot{{\phi }_{M}}\end{array}\right)$$

where $j$ signifies the integer number of the vertical grids with $J$ being its largest grid number; $M$ is the greatest number of the vertical mode. In the equation above, we used the simplified representation for the derivative function: $\dot{{\phi }_{n}}=\frac{\partial {\phi }_{n}}{\partial z}$.

## Supplementary Information

Supplementary Figures.

## Data Availability

Output data of the GPV reanalysis model developed by the Japan Meteorological Association can be downloaded from the website below: http://database.rish.kyoto-u.ac.jp/arch/jmadata/data/gpv/original. The rest of dataset is available upon reasonable request. Correspondence and request for the materials should be addressed to Y.K.
