# Supplementary material for: Amplification of typhoon-generated near-inertial internal waves observed near the Tsushima oceanic front in the Sea of Japan
Source: Sci Rep. 2023 Jun 2;13:8387. doi: 10.1038/s41598-023-33813-9 (PMC10238437; doi:10.1038/s41598-023-33813-9)
Supplement: Supplementary file 1 — Supplementary Figures. [file 41598_2023_33813_MOESM1_ESM.docx]

Supplementary materials

**Amplification of typhoon-generated near-inertial internal waves observed near the Tsushima Oceanic Front in the Sea of Japan**

by Kawaguchi, Y., Yabe, T., Senjyu, and T., Sakai, A.


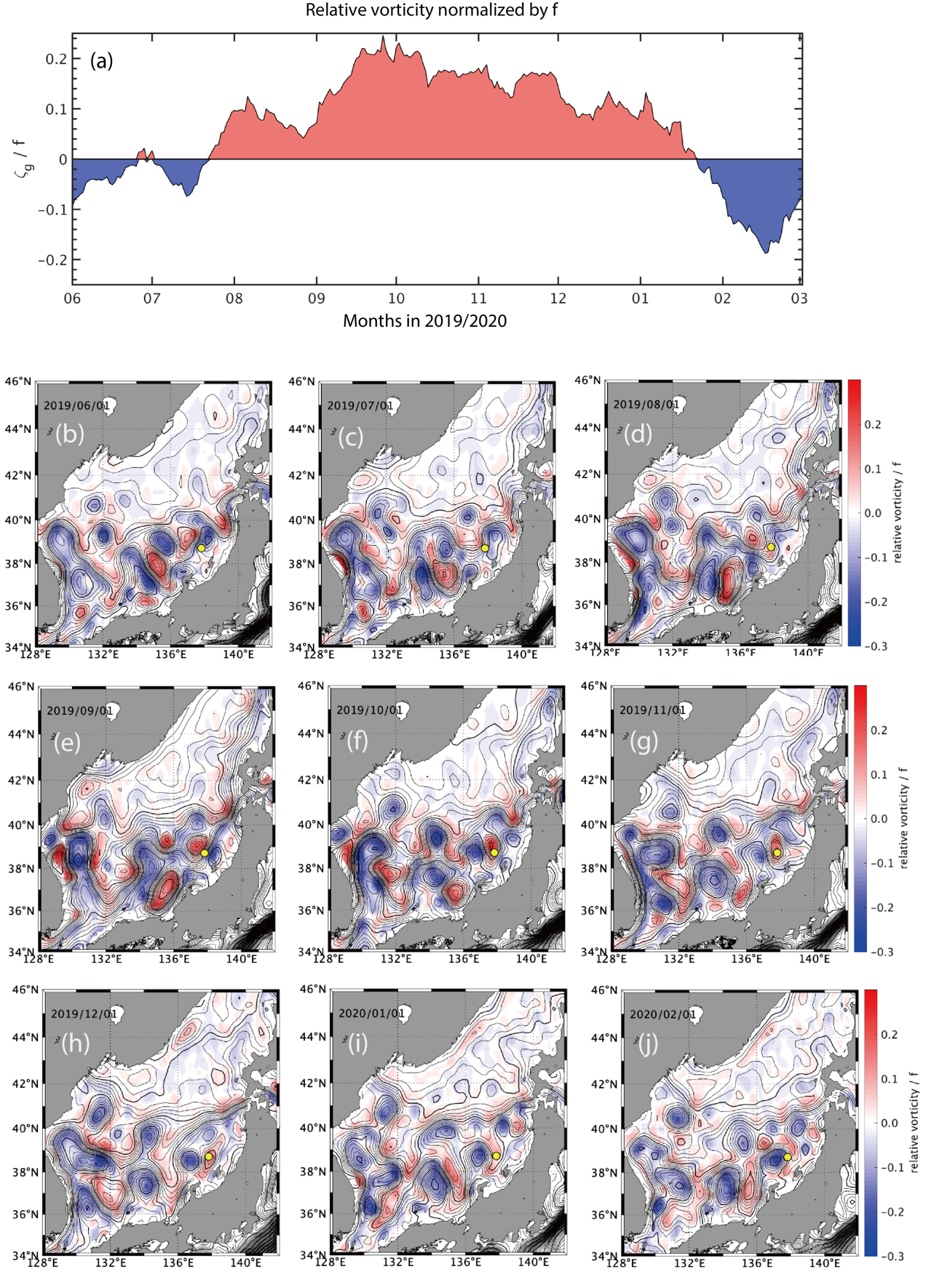


Fig. S1: Time evolution of SSH-based RVA normalized by $\boldsymbol{f}$: (a) monthly timeseries; (b-j) snapshots of horizontal distribution on Day 1 of each month, where yellow dots show the location of the FATO mooring. In (b-j), black contours show SSH with a constant interval of 2 cm.


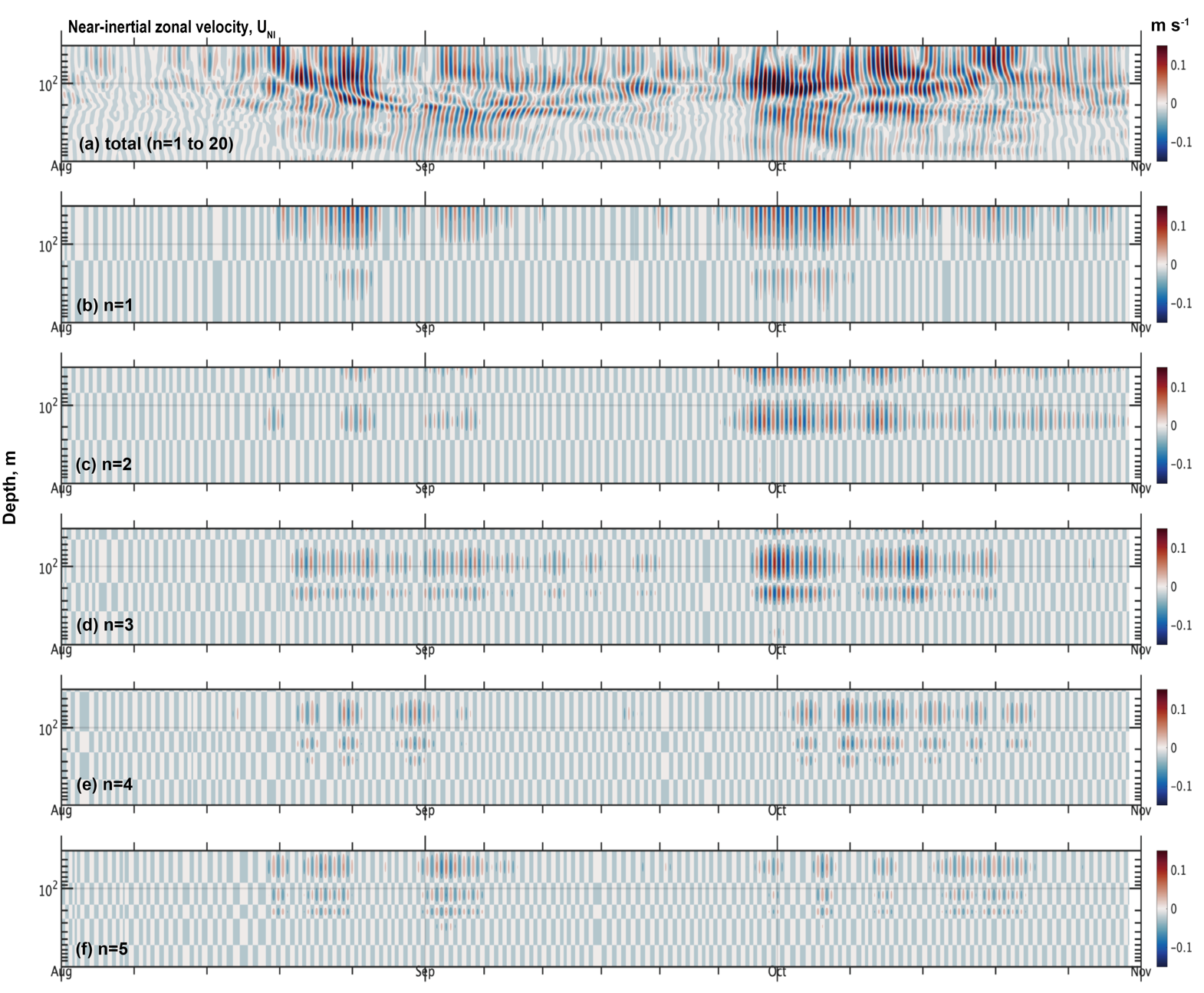


Fig. S2: Modal decomposition of near-inertial zonal velocity: (a) reconstructed $\boldsymbol{U}_{\boldsymbol{NI}}$ with modes $\boldsymbol{n}$ = 1 to 20; (b-f) contributions from the first fifth mode.
